# Target-Mediated Brain Tissue Binding for Small Molecule Inhibitors of Heat Shock Protein 90

**DOI:** 10.3390/pharmaceutics12111009

**Published:** 2020-10-22

**Authors:** Lassina Badolo, Kenneth Thirstrup, Søren Møller Nielsen, Ask Püschl, Thomas Jensen, Steve Watson, Christoffer Bundgaard

**Affiliations:** 1 Translational DMPK, H. Lundbeck A/S, 2500 Copenhagen-Valby, Denmark; CHBG@lundbeck.com; 2 Neurodegeneration, H. Lundbeck A/S, 2500 Copenhagen-Valby, Denmark; kth@Orphazyme.com; 3 Molecular Screening and Pharmacology, H. Lundbeck A/S, 2500 Copenhagen-Valby, Denmark; SMN@Lundbeck.com; 4 Medicinal Chemistry, H. Lundbeck A/S, 2500 Copenhagen-Valby, Denmark; askpuschl@gmail.com (A.P.); Thje@lundbeck.com (T.J.); Steve.Watson@SoseiHeptares.com (S.W.)

**Keywords:** HSP90, small molecule binding brain tissues, target-mediated brain distribution

## Abstract

Drug distribution in the brain is generally associated with an affinity for fatty brain tissues and therefore known to be species- and concentration-independent. We report here the effect of target affinity on brain tissue binding for 10 small molecules designed to inhibit brain heat shock protein 90 (HSP90), a widespread protein whose expression is 1–2% of total cytosolic proteins in eucaryotes. Our results show that increasing the test item concentrations from 0.3 to 100 µM increased the unbound fraction 32-fold for the most potent molecules, with no change for the inactive one (1.1 fold change). Saturation of HSP90 led to normal concentration-independent brain tissue binding. In vivo pharmacokinetics performed in rats showed that the overall volume of distribution of compounds is correlated with their affinity for HSP90. The in vitro binding and in vivo pharmacokinetics (PK) performed in rats showed that small molecule HSP90 inhibitors followed the principle of target-mediated drug disposition. We demonstrate that assessing unbound fractions in brain homogenate was subject to HSP90 target interference; this may challenge the process of linking systemic-free drug concentrations to central nervous system unbound concentrations necessary to establish the proper pharmacokinetics/pharmacodynamics (PK/PD) relation needed for human dose prediction.

## 1. Introduction

According to the principles of the free drug hypothesis, only the unbound (free) drug at the site of action exerts a pharmacological effect. In a central nervous system (CNS) setting, several studies [1,2] have confirmed this hypothesis, demonstrating that the determination of the unbound concentration of a CNS drug in the brain is paramount to understanding the link PK/PD. To this end, free brain concentrations are typically estimated by multiplying total concentrations from entire brains (rodents) or brain sections (human—surgical leftover from clinics) by unbound fractions of the drug in brain tissue determined in vitro. Indeed, in vitro assessment of the binding of drug candidates to brain tissue (unbound fraction in brain) has become routine in drug discovery [3] and many protocols have been developed to perform these studies, the most commonly used being equilibrium dialysis using brain homogenate from rodent.

When robust in vivo pharmacology models are available, determination of unbound brain concentrations at efficacious doses becomes important to relate in vitro potency to in vivo efficacy.

Furthermore, the combination of unbound fractions in plasma/blood and brain homogenate can be used to calculate the ratio of free concentrations in brain relative to plasma (or blood) in vivo; this ratio at steady state is defined as *Kp*,*uu* (*Cu*__Brain/_*Cu*__Plasma_). This ratio expresses the extent of brain disposition, the ability of a drug to freely pass the blood–brain barrier (when *Kp*,*uu* is close to unity) and allows for predicting the human efficacious dose, taking into account the difference between systemic and central exposures.

Binding to plasma or blood proteins varies between species and is associated with the affinity of a drug candidate for albumin or α1-glycoprotein acid; however, binding to brain tissues has been associated with non-specific binding to brain fatty tissues and is thus mainly driven by a compound’s lipophilicity and therefore species-independent [4]. Many authors have indeed reported that binding to brain tissues is species-independent [3,5,6]. Because of the high concentration of lipid in the brain—around 11% [7,8]—binding to brain adipose tissues is also expected to be concentration-independent within a large range of concentrations.

In CNS settings, although drug molecules bind to their target proteins, the level of expression of these targets is typically vastly below the amount of fat tissue responsible for non-specific binding; thus, binding of a compound to the CNS receptors has little measurable effect on the overall non-specific binding to brain tissues which is essentially controlled by the non-specific binding to brain fat. 

In the present paper, we present brain tissue binding data for inhibitors of heat shock protein 90 (HSP90), a widespread and highly expressed protein constituting around 1–2% of cytosolic proteins in eukaryotes [9,10]. The level of HSP90 is sufficiently high that it affects the level of non-specific binding of HSP90 inhibitors to brain tissues. This phenomenon has previously been reported for HSP90 inhibitors in a peripheral setting and termed “target-mediated drug disposition” [11]. Observation of this phenomenon in a CNS setting is reported here. The relation between affinity for HSP90 and binding to brain tissues will be discussed as well as its consequence on the development of small molecule inhibitors of HSP90 in a CNS setting.

## 2. Materials and Methods

### 2.1. Affinity for HSP90

Affinity for HSP90 was assessed through Ki determination using scintillation proximity assay (SPA), with recombinant HSP90β and [^3^H]-17AAG (17-Allylamino-17-demethoxygeldanamycin) as tracer. The details of determination are available in [12].

### 2.2. Determination of Unbound Fractions Using Equilibrium Dialysis

Brain tissue from Naval Medical Research Institute (NMRI) mice was homogenized in phosphate buffer (1 g: 2 mL) and stored in −80 °C freezer before use. For dialysis experiment, 150 µL of brain homogenate (fresh blood collected on EDTA-K2 and diluted 1:1 with phosphate buffer or plasma) was added to one compartment of a dialysis plate (assembled according to the manufacturer instructions with a dialysis membrane between the 2 compartments) against 150 µL of phosphate buffer pH 7. The test compound was added to the compartment with brain homogenate (or plasma) and the plate was incubated in an incubator with a 5% CO_2_ and rotating at 150 rpm for 5 h. At the end of the incubation time, aliquots of 90 µL of samples were removed from both the buffer and the brain homogenate compartments. The aliquots were added to 120 µL of acetonitrile containing the internal standard. The mixture was centrifuged at 4000 rpm for 10 min and the supernatant was collected for LC-MS/MS analysis. All equilibrium dialysis studies were performed in triplicate at each tested concentration.

Binding to brain tissues was assessed using 6 different concentrations of test items ranging from 0.3 to 100 µM. Warfarin was used as control.

### 2.3. Data Analysis

Determination of fractions unbound was conducted according to the equations below:

*fu* = *C*receiver (*C*_T0_ − *C*receiver) (1)

In brain homogenate (blood or plasma):

(2) $$\%\;Undiluted\;fu=100\;\times \frac{1/D}{\frac{1}{fu}-1+1/D}$$

(3) $$\%\;Recovery=100\;\times \;\frac{Creceiver\;+Cdonor}{C_{T0}}$$

where

*C*_receiver_ = free compound concentration on the buffer side of the membrane

*C*_donor_ = total compound concentration on the plasma, blood or brain side of the membrane

*C*_T0_ = total compound concentration as determined before analysis

*fu* is the free fraction measured following a known fold dilution of brain tissue.

Dilution factor (*D*) is determined as 3 in this assay.

### 2.4. Animals

Male, drug-naïve Sprague–Dawley rats (250–320 g obtained from Charles River, Sulzfeld Germany) were used for all in vivo studies. The rats were housed pair-wise in temperature and humidity controlled environment under a 12-h light/dark cycle (lights on at 06:00 a.m.). Food and tap water were freely available in the home cage. The rats had a minimum of 5 days’ adaptation in the animal facility prior to the initiation of experiments. Ethical permission for the procedures used in this in vivo study was granted by the Danish Animal Experiments Inspectorate and animal procedures were performed in compliance with Directive 2010/63/EU of the European Parliament and of the Council and with Danish Law and Order regulating animal experiments (LBK no 253, 8 March 2013 and BEK no 88, 30 January 2013). 

### 2.5. In Vivo Rat Pharmacokinetics Studies

All in vivo PK studies were performed after intravenous (iv) administration of 1 mg/kg test compound. All drugs were dosed in solution at a volume of 1 mL/kg bodyweight using appropriate vehicles. Serial blood samples (200 μL) were taken from the tail vein at regular time intervals to obtain plasma concentration–time courses for each individual animal. Blood samples were collected in EDTA-coated tubes and centrifuged for 10 min at 4 °C, after which plasma was drawn off.

Plasma samples (25 µL) were precipitated by adding 150 µL of 100% acetonitrile containing 5 ng/mL of internal standard. Samples were centrifuged at a temperature of 4 °C at 6200× *g* for 20 min and 100 μL of supernatants were diluted with equal volume of water. Then, 10 µL of the mixture was injected into the LC-MS. Concentrations were calculated from standard curves covering the relevant concentration ranges.

### 2.6. Data Analysis

PK parameters were calculated by non-compartmental analysis of the individual plasma concentration–time courses using WinNonlin (Certara Corp, Vers. 5.2., Princeton, NJ, USA). All results were expressed as mean values (±sem).

### 2.7. LC-MS Analysis of In Vitro Samples and In Vivo Samples

#### 2.7.1. Bioanalysis of In Vitro and In Vivo Samples

The samples were analyzed with liquid chromatography tandem mass spectrometry (LC-MS) using a Shimadzu LC-20-AD (Kyoto, Japan) (ACE 5 Phenyl 2.1 × 50 mm ACE-125-0502 column) coupled to either a Sciex API4000 or API6500+ mass spectrometer (Framingham, MA, USA).

The mobile phases used were A: 0.1% formic acid in water and B: 0.1% formic acid in acetonitrile. The following gradient was applied at a flow rate of 700 µL/min: time (min)/% of mobile phase B: 0.01 min/5%; 0.8 min/95%; 1 min/95%; 1.01 min/5%; 1.2 min/5%.

All compounds were analyzed on MS in positive ionization mode using multiple reaction monitoring mode. MS settings are compiled in the table below:



**MS/MS Transitions (m/z)**

**CE (eV)**

**Inject Volume (μL)**
Lu1394.2 > 256.13510Lu2380.1 > 335.13510Lu3400.2 > 337.13510Lu4299.3 > 162.03510Lu5365.2 > 216.12010Lu6423.3 > 353.23510Lu7284.3 > 162.05010Lu8408.2 > 338.3358Lu9319.1 > 120.1358Lu10299.3 > 120.1352

#### 2.7.2. LC Methods for In Vivo PK Studies

Lu2: Acquity UPLC/API4000: Column: Waters XSELECT CSH XP C18, 2.5 µm, 2.1 × 50 mm. Mobile phases A: 2 mM ammonium acetate in water; B: 0.1% formic acid in 95% acetonitrile/5% water. Gradient (time/% B): 0 min/30%, 1.2 min/90%, 1.4 min/90%, 1.41 min/30%, 1.5 min/30%.

Lu3: Acquity UPLC/API4000: Column: BEH C18, 1.7 µm, 2.1 × 50 mm. Mobile phases A: 0.1 formic acid in 95% acetonitrile/5% water; B: 0.1% formic acid in 95% acetonitrile/5% water. Gradient (time/% B): 0 min/30%, 1.2 min/80%, 1.4 min/80%, 1.41 min/30%, 1.5 min/30%.

Lu4: Acquity UPLC/API4000: Column: BEH C8, 2.1 × 50 mm. Mobile phases A: 0.1% ammonium acetate in water B: 0.1% ammonia in water (25%)/acetonitrile (75%). Gradient (time/% B): 0 min/2%, 0.01 min/2%, 1.5 min/95%, 2.0 min/95%, 2.2 min/2%, 3.0 min/2%.

Lu8: Acquity UPLC/API4000: Column: BEH C8, 2.1 × 50 mm. Mobile phases A: 0.1% formic acid in water; B: 0.1% formic acid in acetonitrile. Same gradient as for Lu4.

## 3. Results

### 3.1. Inhibition of HSP90 Protein

Of the 10 compounds tested, affinity for HSP90 (Table 1) ranged from 0.7 nM for the most potent compound (Lu2) to almost inactive compounds with Ki higher than 10,000 nM (Lu1 and Lu5).

### 3.2. Unbound Fraction Determination in Mouse Brain Homogenate

The results expressed as free fractions showed (Table 2) a change in binding to brain tissues leading to an increase in unbound fractions from 0.3 or 1 to 100 µM for a number of compounds (Lu2, Lu3, Lu4, Lu6, Lu8, Lu9 and Lu10). For Lu1, Lu5 and Lu7, no significant change in free fraction was observed from 0.3 to 100 µM (ratio of unbound fractions 100 µM/0.3 µM < 2). Recoveries were all in the acceptable range of 80–120%.

### 3.3. Effect of HSP90 Inhibition on the Free Fractions in Brain Homogenate

Binding to brain tissues was assessed for all test compounds (except for Lu2) at three concentrations (1, 10 and 30 µM) in the presence of 100 µM of Lu2 (Ki = 0.72 nM). The results showed that none of the test compounds displayed a change in binding at the three concentrations tested in the presence of Lu2. The ratios of free fraction at 30 µM over 1 µM remained close to unity (see Table 3).

### 3.4. Relation between Binding to Brain Tissues and Affinity for HSP90

**Figure 1:**
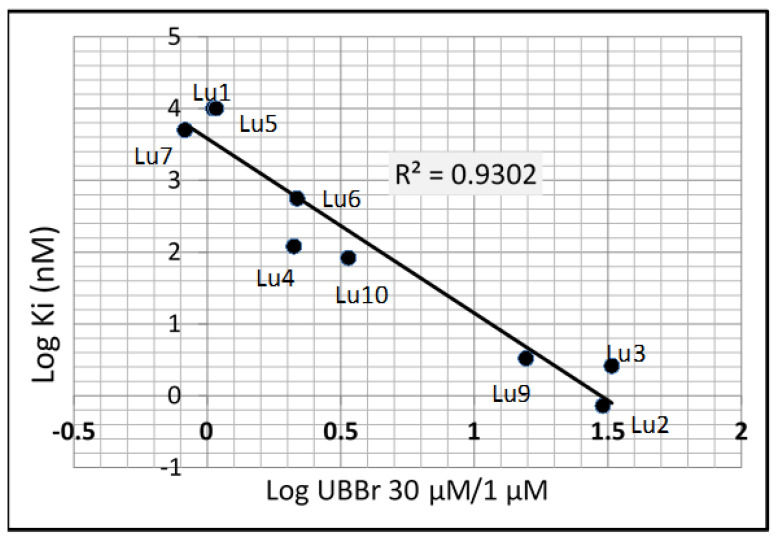
Correlation between change in brain tissue binding and affinity for HSP90. Correlation between shift in brain tissue binding (expressed as ratios of unbound fractions at 30 μM over fractions determined at 1 μM) and affinity for HSP90.

In order to establish a relation between affinity for HSP90 and changes in unbound fractions in brain homogenate, ratios of free fractions at 30 µM over 1 µM were plotted against Ki for HSP90 (Figure 1). The results showed that there is a linear correlation (r^2^ = 0.94) between the shift in unbound fraction from 30 and 1 µM and the affinity of the Ki values. Compounds with highest affinity for HSP90 (lowest Ki) have the highest change in unbound fractions, while the ratio is close to unity for inactive compounds. The highest increase in free fraction (30-fold) was observed for the most potent compound (Lu2) with Ki of 0.72 nM.

### 3.5. Binding to Plasma and Blood Tissues

Unbound fractions in mouse plasma and blood were assessed at 1 µM for seven of the above compounds, with affinities ranging from 0.7 to 5000 nM. In all cases, the unbound fractions in plasma were very similar to that of blood (see Table 4). This means that HSP90 inhibitors do not bind more strongly to erythrocytes and other blood cells than to plasma protein, suggesting either no or a marginal expression of HSP90 in blood cells. Plasma protein data (unbound fractions) ranged from 1.8% to 51%. Recoveries were all in the acceptable range of 80–120%.

### 3.6. PK in Rats: Overview of PK Parameters

The four inhibitors profiled in vivo have HSP90 affinities ranging from 0.2 to 120 nM. The PK data showed (see Table 5) that all compounds tested have a systemic clearance around 25% of liver blood flow. The T½ ranged from 0.3 to 5.1 h, due to the significant difference in Vss (from 0.6 to 7.1 L/kg).

### 3.7. Relation between HSP90 Target Affinity and Compound Distribution in Rats In Vivo

**Figure 2:**
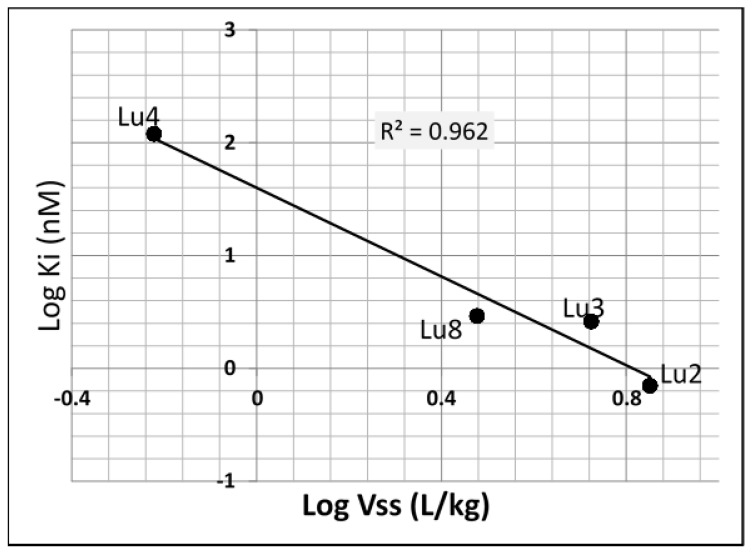
Correlation between affinity for HSP90 and volume of distribution in rats.

For the four compounds studied, rats were injected IV with 1 mg/kg test compound; blood samples were then collected for exposure and pharmacokinetics parameter determination. The results showed that all compounds had low IV clearance (CL) at around 1 L/h/kg. Despite similarity in structures, distribution volumes (Vss) were different between the compounds ranging from 0.6 to 7.1 L/kg. The volume was highest for the most potent compound and lowest for the less potent one. A linear correlation (r^2^ = 0.96) was observed between affinity for HSP90 and the distribution volume (Figure 2).

## 4. Discussion

The need to assess the effective free concentration of CNS drugs in the brain has made the assessment of binding to brain tissues a routine technique for CNS drug discovery [3]. Small molecule binding to brain tissues, assessed mostly using equilibrium dialysis or ultrafiltration techniques, is species- and concentration-independent. It is commonly understood that only lipophilicity drives the binding of drug molecules to the fatty brain tissues.

Our report here shows that all these paradigms hold true as long as the target protein expression in the brain is far below the concentration of fatty brain tissues and much lower than the drug concentrations. HSP90 represents 1–2% of all cytosolic proteins in eucaryotes [9,10]; for such highly expressed proteins, we show that affinity for the target has an effect on the overall binding to brain tissues. Increasing HSP90 inhibitor concentrations from 0.3 to 100 µM in the incubation medium led to a significant increase in unbound fractions for potent inhibitors, while the inactive ones did not show any change in unbound fractions, supporting the fact that the observed change was associated with the affinity of compounds to HSP90 protein. A linear correlation was indeed observed between the change in free fractions and Ki (Figure 1). 

Preincubation of brain homogenate with 100 µM of Lu2, the most potent of the tested compounds (Ki = 0.72 nM), was designed to saturate HSP90; therefore, subsequent addition of less potent compounds would only/mostly allow non-specific binding to brain fatty tissues and display “normal” behavior, with no concentration-dependent change in free fractions. This corresponds to our observation when these compounds were tested at 1, 10 and 30 µM in the presence of 100 µM of Lu2 where none of the compounds showed changes in unbound fractions. This supports our initial observation that the observed concentration-dependent change in unbound fraction for HSP90 inhibitors was associated with their interaction with the HSP90 target present in brain homogenate used to assess brain unbound fractions (Table 3).

Having established this link makes it challenging to establish robust CNS PK/PD relationships for HSP90 inhibitors. The classical way of thinking of compound distribution from blood to brain based on a two-compartment model with blood on one side and brain on the other, leaving freely the unbound drug to equilibrate between brain and blood, should be revised and extended to include a third compartment which is the target protein, HSP90 itself.

Whilst BBB permeability, affinity for plasma proteins and affinity for brain fatty tissues control distribution of drugs between the brain from blood for most drugs, when targeting HSP90 in the brain, affinity for HSP90 will play an additional role in controlling the equilibrium between the fatty brain tissues and the abundant HSP90 protein in the brain. In such circumstances, and in the absence of steady-state conditions (achieved by infusion over a sufficient period of time), unbound drug concentrations in the brain are expected to be influenced by two equilibrium states dependent on time and total drug concentration. This renders a proper understanding of PK/PD for HSP90 central drugs complex and may compromise the success of projects aiming at inhibiting HSP90 in CNS.

As a ubiquitous protein with global expression of around 1% to 2% of the entire cytosolic body proteins in eucaryotes, one would expect that the affinity of drugs for HSP90 may impact their total distribution, with HSP90 functioning as an extra compartment being able to subtract a significant proportion of drug from blood for HSP90 high affinity molecules. This was observed for the four analog molecules profiled in vivo. Despite the structural similarity and the almost identical clearance observed in rats, the volume of distributions ranged from 0.6 to 7.1 L/kg, with a significant consequence on the half-lives of the compounds. A linear correlation was established between affinity (Ki) for HSP90 and volume of distribution. This supports the finding made by Yamazaki et al. [11] testing six compounds in vivo. They concluded that small molecule HSP90 inhibitors followed the principles of target-mediated drug disposition [11]. In blood, although HSP90 may be expressed in various cells, our data showed no significant difference between plasma and blood binding for high affinity HSP90 inhibitors, suggesting that HSP90 expression in blood cells is relatively low. However, it cannot be excluded that binding to HSP90 contributes to the observed plasma or blood free fractions data shown in Table 4. 

HSP90 is known to be involved in many biological processes and has been identified as a potential target in many indications in cancer therapies [13], CNS diseases [14,15], inflammatory diseases in general [16] or rheumatic diseases [17]. As such, this target is the focus of many research programs aiming at identifying small molecule inhibitors of HSP90.

The very high expression of HSP90 throughout the entire body makes this target challenging in small molecule drug discovery, as targeting a specific tissue may be difficult to achieve. In addition, the target-mediated drug disposition profile requires a higher level of understanding of the drug PK/PD relationships that are needed to support optimal selection of drug candidates. This is in line with the conclusions made in two reviews [18,19].

Our results show that assessing unbound fractions in brain homogenate may be subject to target interference for CNS targets whose expression is as high as HSP90 and challenge the process of development of small molecule inhibitors of brain HSP90. 

## Figures and Tables

**Table 1:** Affinity (Ki, nM) for test items for HSP90.

Test Compound	MW (g/mol)	cLogP	TPSA	H_Don	H_Ac	Ki (nM)
Lu1	393	1.9	103	2	6	>10,000 *
Lu2	379	1.8	103	2	6	0.72 ± 0.4
Lu3	399	3.5	86	2	4	2.6 ± 0.9
Lu4	298	1.6	91	1	6	120 ± 33
Lu5	364	2.3	91	1	6	>10,000 *
Lu6	423	3.1	85	2	5	560 (540; 572)
Lu7	283	0.9	88	1	6	5000 (4566; 5577)
Lu8	408	3.5	72	2	4	2.9 ± 0.7
Lu9	319	2.2	92	1	6	3.3 ± 1.6
Lu10	298	1.4	91	1	6	83 ± 43

Binding affinities of HSP90 inhibitors were determined from competition binding experiments between [^3^H]-17AAG and the compound of interest. The results are mean ± SD of n = 2–22 (individual numbers are shown for n = 2). Correlation between cellular potency and HSP90 target occupancy was established and published [12]. * For correlation determination, these numbers were set to 10,000 nM. TPSA (topological polar surface area), H_Don (number of hydrogen bond donors), H_Ac (number of hydrogen bond acceptors).

**Table 2:** Concentration-dependent change in binding to brain tissues for HSP90 inhibitors.

	Unbound Fraction (%) in Mouse Brain Homogenate at Different Concentrations of Test Compounds					
Test Item	0.3 µM	1 µM	3 µM	10 µM	30 µM	100 µM
Warfarin	8.6 ± 0.2	11.2 ± 0.1	14.3 ± 0.5	14.3 ± 0.6	16.8 ± 0.6	17.9 ± 0.9
Lu1	4.6 ± 0.4	4.8 ± 0.3	5.7 ± 0.3	3.8 ± 0.5	5.0 ± 0.8	6.9 ± 0.4
Lu2	NA	0.1 ± 0.01	2.4 ± 0.02	2.8 ± 0.4	3.6 ± 0.2	3.2 ± 0.6
Lu3	NA	0.1 ± 0.1	1.1 ± 0.02	2.0 ± 0.1	2.1 ± 0.2	2.2 ± 0.1
Lu4	6.5 ± 0.3	41.8 ± 7.3	20.4 ± 1.8	11.3 ± 0.4	23.9 ± 2.6	21.9 ± 5.5
Lu5	2.6 ± 0.2	2.6 ± 0.2	2.6 ± 0.1	2.7 ± 0.2	2.8 ± 0.2	2.1 ± 0.1
Lu6	1.6 ± 0.03	2.7 ± 0.2	5.3 ± 0.2	5.3 ± 0.1	5.9 ± 0.2	7.8 ± 0.5
Lu7	9.0 ± 0.6	9.1 ± 0.1	8.9 ± 1.6	10.2 ± 1.3	7.5 ± 0.8	9.7 ± 0.3
Lu8	0.02 ± 0.01	0.3 ± 0.02	0.9 ± 0.03	0.8 ± 0.1	0.5 ± 0.03	0.8 ± 0.1
Lu9	0.2 ± 0.004	0.8 ± 0.1	9.2 ± 0.8	17.8 ± 3	12.3 ± 0.5	15.3 ± 1.9
Lu10	2.7 ± 0.1	4.1 ± 0.1	12.0 ± 0.5	15.0 ± 0.6	13.8 ± 1.4	20.9 ± 1.6

Unbound fraction (%) of compounds assessed in mouse brain homogenate. Results are mean ± SD from n = 3 determinations.

**Table 3:** Effect of HSP90 saturation with 100 µM of Lu2 on the concentration-dependent change in binding to brain tissues for HSP90 inhibitors.

Test Item	Ratio (30 µM/1 µM) Unbound Fractions in Brain	Ratio (30 µM/1 µM) Unbound Fractions in Brain after Saturation of HSP90 with 100 µM Lu2
Warfarin	1.5	1.4
Lu1	1.0	0.9
Lu3	21.0	1.0
Lu4	0.6	0.7
Lu5	1.1	1.1
Lu6	2.2	1.0
Lu7	0.8	0.7
Lu8	1.7	1.3
Lu9	15.4	1.1
Lu10	3.4	0.8

The use of high concentration of the highly potent Lu2 was designed to saturate HSP90 in brain homogenate; therefore, the values of unbound fractions assessed in presence of 100 µM of Lu2 correspond solely to the non-specific binding of the compounds to brain fatty tissues.

**Table 4:** Binding to plasma and blood proteins for HSP90 inhibitors.

	Free Fractions (%)			
	Mouse Plasma Free Fraction (UBP)	Mouse Blood Free Fraction (UBB)	Ratio UBP/UBB	Ki (nM)
Lu2	6.7 ± 0.2	6.5 ± 0.3	1.03	0.72
Lu3	4.3 ± 1.1	4.9 ± 0.3	0.88	2.6
Lu4	51 ± 4.2	29.9 ± 2.8	1.70	120
Lu7	29 ± 0.9	15.7 ± 2.2	1.85	5000
Lu8	1.8 ± 0.2	3.0 ± 0.1	0.59	2.9
Lu9	23.8 ± 2.2	15.9 ± 1.4	1.50	3.3
Lu10	10.1 ± 0.1	9.6 ± 0.8	1.05	83

**Table 5:** Overview of PK parameters in rats after IV administration of 1 mg/kg test compound.

	Ki (nM)	CL (L/h/kg)	Vss (L/kg)	T_½_ (h)	AUC_0–∞_ (ng.h/mL)
Lu2	0.72	1.1 ± 0.08	7.1 ± 2.0	5.1 ± 1.3	924 ± 73
Lu3	2.6	1.2 ± 0.2	5.3 ± 1.5	3.2 ± 0.3	889 ± 174
Lu4	120	1.2 (1.0; 1.4)	0.6 (0.5; 0.6)	0.3 (0.3; 0.4)	877 (1040; 713)
Lu8	2.9	1 (1; 1)	3 (3.1; 3)	2.1 (2.1; 2)	828 (831; 825)
